# A131 OUTCOMES AND COMPLICATIONS OF THERAPEUTIC ERCP IN DIFFERENT AGE GROUPS

**DOI:** 10.1093/jcag/gwad061.131

**Published:** 2024-02-14

**Authors:** E Hazan, C Na, A Zarrin, A Mokhtar, K Khalaf, N Gimpaya, M Meleka, D Tham, S P Abal, W Mhalawi, D Chopra, S Gupta, S Jugnundan, N Calo, C Teshima, J Mosko, G May, S Grover

**Affiliations:** University of Toronto Temerty Faculty of Medicine, Toronto, ON, Canada; St. Michael's Hospital, Toronto, ON, Canada; The University of British Columbia Faculty of Medicine, Vancouver, BC, Canada; University of Toronto Temerty Faculty of Medicine, Toronto, ON, Canada; Division of Gastroenterology, St Michael's Hospital, Toronto, ON, Canada; St. Michael's Hospital, Toronto, ON, Canada; St. Michael's Hospital, Toronto, ON, Canada; Faculty of Health Sciences, McMaster University Faculty of Health Sciences, Hamilton, ON, Canada; St. Michael's Hospital, Toronto, ON, Canada; St. Michael's Hospital, Toronto, ON, Canada; St. Michael's Hospital, Toronto, ON, Canada; University of Toronto Temerty Faculty of Medicine, Toronto, ON, Canada; University of Toronto Temerty Faculty of Medicine, Toronto, ON, Canada; St. Michael's Hospital, Toronto, ON, Canada; St. Michael's Hospital, Toronto, ON, Canada; St. Michael's Hospital, Toronto, ON, Canada; St. Michael's Hospital, Toronto, ON, Canada; St. Michael's Hospital, Toronto, ON, Canada

## Abstract

**Background:**

Endoscopic Retrograde Cholangiopancreatography (ERCP) is a vital procedure for hepatobiliary disease management. Though it boasts high overall success rates, the impact of patient age on outcomes remains uncertain, especially amongst elderly patients. With an aging population, it is crucial to determine the efficacy and safety of ERCP in different age groups.

**Aims:**

This study assesses ERCP success and complication rates across age groups. Our objective is to investigate whether ERCP remains a reliable and safe procedure for patients of all ages, particularly the elderly.

**Methods:**

A database with retrospective data from consecutive patients undergoing ERCP at a Canadian tertiary care centre between 2011 and 2020 was utilized. Baseline patient characteristics and indications for ERCP were collected. Outcomes included procedural success, complications, and procedure duration. Patients were categorized into four age groups: ampersand:003C40, 40-65, 65-80, and ampersand:003E80, with a specific sub-analysis for patients ampersand:003C80 and ampersand:003E80.

**Results:**

This study included 6132 patients. Significant disparities were observed in comorbid conditions (pampersand:003C0.0001), ASA status (pampersand:003C0.0001), and the use of anticoagulants or antiplatelet agents (pampersand:003C0.0001). The most common indications for ERCP were gallstones (45.1%), jaundice (19.2%), and ductal lesions (12.8%), with no significant variation among the age groups (p=0.5983). There was a significant difference in procedural success (p=0.0005), with declining success in older age groups (ampersand:003C40 = 91.3%, 40-65 = 87.4%, 65-80 = 86.2%, ampersand:003E80 = 84.9%), and this difference remained significant in the ampersand:003C80 and ampersand:003E80 sub-analysis (p=0.0133). While mean procedure duration was longer in older age groups (pampersand:003C0.0001), incidence of complications did not vary across all groups (p values [EH1] = 0.1532-0.7622) or patients ampersand:003C80 and ampersand:003E80 (p= 0.0769-0.7128).

**Conclusions:**

ERCP maintains a high success rate and safety profile across all age groups; however, success rates decline and procedure times increase significantly in older patients.

ERCP outcomes across age groups

**Funding Agencies:**

None

**Table:** 

Outcomes	ampersand:003C40	40-65	65-80	80+	P-value (ChiSq)
Overall, Success (n)	91.26% (637)	87.36 (1873)	86.23% (1697)	84.87% (1122)	0.0005
Immediate Bleeding (n)	3.32% (23)	4.43% (94)	4.26% (83)	5.33% (70)	0.1979
Delayed Bleeding (n)	0.29% (2)	0.10% (2)	0.21% (4)	0.31% (4)	0.3912
Pancreatitis (n)	0.72% (5)	0.28% (6)	0.20% (4)	0.15% (2)	0.1532
Perforation (n)	0.29% (2)	0.14% (3)	0.15% (3)	0.23% (3)	0.7622
Abdominal Pain (n)	0.72% (5)	0.89% (19)	0.61% (12)	0.45% (6)	0.4850
Mean Procedure Duration (Minutes)	28.87±23.51	32.99±20.76	33.89±19.70	34.30±17.54	ampersand:003C0.0001

